# Prevalence and Load of Cervical Ureaplasma Species With Respect to Intra-amniotic Complications in Women With Preterm Prelabor Rupture of Membranes Before 34 weeks

**DOI:** 10.3389/fphar.2022.860498

**Published:** 2022-03-31

**Authors:** Marian Kacerovsky, Rudolf Kukla, Radka Bolehovska, Pavel Bostik, Jana Matulova, Jan Mls, Jaroslav Stranik, Bo Jacobsson, Ivana Musilova

**Affiliations:** ^1^ Department of Obstetrics and Gynecology, University Hospital Hradec Kralove, Faculty of Medicine in Hradec Kralove, Charles University, Hradec Kralove, Czech Republic; ^2^ Biomedical Research Center, University Hospital Hradec Kralove, Hradec Kralove, Czech Republic; ^3^ Institute of Clinical Biochemistry and Diagnostics, Faculty of Medicine in Hradec Kralove, University Hospital Hradec Kralove, Charles University, Hradec Kralove, Czech Republic; ^4^ Institute of Clinical Microbiology, University Hospital Hradec Kralove, Charles University, Faculty of Medicine in Hradec Kralove, Hradec Kralove, Czech Republic; ^5^ Department of Social Medicine, Charles University, Faculty of Medicine in Hradec Kralove, Hradec Kralove, Czech Republic; ^6^ Department of Obstetrics and Gynecology, Institute of Clinical Science, Sahlgrenska Academy, University of Gothenburg, Gothenburg, Sweden; ^7^ Region Västra Götaland, Sahlgrenska University Hospital, Department of Obstetrics and Gynecology, Gothenburg, Sweden; ^8^ Department of Genetics and Bioinformatics, Domain of Health Data and Digitalization, Institute of Public Health, Oslo, Norway

**Keywords:** microbial invasion of the amniotic cavity, genital *mycoplasma*, intra-amniotic inflammation, non-invasive sample, preterm delivery

## Abstract

**Objectives:** To determine the prevalence and load of *Ureaplasma* spp. DNA in the cervical fluid of women with singleton pregnancies complicated by preterm prelabor rupture of membranes (PPROM) with respect to intra-amniotic infection, sterile intra-amniotic inflammation, and colonization of the amniotic fluid.

**Methods:** A total of 217 women with PPROM between gestational ages 24 + 0 and 33 + 6 weeks were included in this study. Paired amniotic and cervical fluid samples were collected at the time of admission via transabdominal amniocentesis and using a Dacron polyester swab, respectively. Microbial invasion of the amniotic cavity was diagnosed using a combination of culture and molecular biology methods. Intra-amniotic inflammation was determined based on the concentration of interleukin-6 in the amniotic fluid. Based on the presence or absence of these conditions, the women were stratified into the following subgroups: intra-amniotic infection (with both), sterile intra-amniotic inflammation (with inflammation only), colonization (with microorganisms only), and negative amniotic fluid (without either). The *Ureaplasma* spp. DNA load in the cervical fluid was assessed using PCR.

**Results:** *Ureaplasma* spp. DNA in the cervical fluid was found in 61% (133/217) of the women. Women with negative amniotic had similar prevalence of *Ureaplasma* spp. DNA in cervical fluid (55%) to those with sterile intra-amniotic inflammation (54%) but lower than those with intra-amniotic infection (73%) and colonization (86%; *p* < 0.0001). Women with negative amniotic fluid had a lower load of *Ureaplasma* spp. DNA in their cervical fluid (median: 4.7 × 10^3^ copies of DNA/ml) than those with intra-amniotic infection (median: 2.8 × 10^5^ copies DNA/ml), sterile intra-amniotic inflammation (median: 5.3 × 10^4^ copies DNA/ml), and colonization (median: 1.2 × 10^5^ copies DNA/mL; *p* < 0.0001).

**Conclusion:** In conclusion, in PPROM at <34 weeks, the presence of intra-amniotic infection, sterile intra-amniotic inflammation, or colonization of the amniotic fluid was associated with a higher prevalence and/or load of *Ureaplasma* spp. DNA in the cervical fluid than the absence of intra-amniotic complications.

## Introduction

Preterm prelabor rupture of the membranes (PPROM) is defined as rupture of the fetal membranes with leakage of amniotic fluid before the onset of regular uterine activity before 37 weeks of gestation (Mercer, 2003; Mercer, 2005). PPROM is one of the “great obstetrical syndromes,” with considerable medical and socio-economic impacts (Romero, 1996; Romero et al., 2006; Di Renzo, 2009; Brosens et al., 2011). PPROM remains under intensive debate among scientists, researchers, and clinicians given the necessity: 1) to fully unravel the underlying pathophysiological mechanisms to make the prevention of PPROM possible (Moore et al., 2020); 2) to understand the underlying mechanisms affecting the interval between PPROM and delivery to optimize the timing of induction of lung maturity (Battarbee et al., 2020); 3) to characterize the causality and consequences of microbial invasion of the amniotic cavity (the presence of microorganisms and/or their DNA in the amniotic fluid) and intra-amniotic inflammation (elevation of inflammatory mediators in the amniotic fluid) (Menon and Richardson, 2017); and 4) to identify risk factors and reliable biomarkers of microbial invasion of the amniotic cavity and intra-amniotic inflammation to enable a individualized therapeutic approach (Stranik et al., 2021).

The presence of microbial invasion of the amniotic cavity and intra-amniotic inflammation complicates approximately 23–41% and 17–58%, respectively, of pregnancies with PPROM (Romero et al., 2015; Kacerovsky et al., 2020a; Kacerovsky et al., 2021b). Based on these two intra-amniotic complications, the following scenarios can occur in PPROM pregnancies: 1) intra-amniotic infection (presence of both), 2) sterile intra-amniotic inflammation (intra-amniotic inflammation only), 3) colonization of the amniotic fluid (microbial invasion of the amniotic cavity only), and 4) negative amniotic fluid (absence of both) (Musilova et al., 2015; Romero et al., 2015).

There is plethora of evidence that *Ureaplasma* spp. are the most common microorganisms recovered from the amniotic fluid obtained from PPROM pregnancies. Therefore, these low-virulence bacteria with sizes comparable to those of viruses represent the most common cause of intra-amniotic infection or colonization of the amniotic cavity in PPROM (Kacerovsky et al., 2021b). Because they are commonly found in the cervical/vaginal niche of women with PPROM, the lower genital tract is considered the main source of amniotic fluid *Ureaplasma* spp.

The presence of *Ureaplasma* spp. in the cervical/vaginal niche in PPROM pregnancies is related to a higher risk of ascension and their subsequent presence in amniotic fluid (Musilova et al., 2016). Nevertheless, there is a shortage of information on whether their prevalence and loads in the cervical niche might be prone to a specific subtype of intra-amniotic complications, such as intra-amniotic infection, sterile intra-amniotic inflammation, and colonization of the amniotic cavity.

To fill this knowledge gap, this study aimed to determine the prevalence of *Ureaplasma* spp. DNA in the cervical fluid of the subgroups of women with PPROM with intra-amniotic infection, sterile intra-amniotic inflammation, colonization of the amniotic cavity, and negative amniotic fluid. The secondary aim was to compare the microbial load of *Ureaplasma* spp. DNA in the cervical fluid among subgroups of women with PPROM. The final aim was to compare the relative abundance of *Ureaplasma* spp. DNA in the cervical fluid among subgroups of women with PPROM.

## Materials and Methods

In this retrospective study, we included pregnant women admitted to the Department of Obstetrics and Gynecology of the University Hospital Hradec Kralove, Czech Republic, between May 2015 and May 2021, who met the following criteria: 1) age ≥18 years; 2) singleton pregnancy; 3) gestational ages between 24 + 0 and 33 + 6 weeks; 4) PPROM; and 5) amniocentesis to assess the intra-amniotic environment. The exclusion criteria were as follows: 1) pregnancy-related complications (e.g., fetal growth restriction, gestational, gestational hypertension, and preeclampsia); 2) chronic medical complications (e.g., pregestational diabetes mellitus and chronic hypertension); 3) congenital or chromosomal fetal abnormalities; 4) signs of fetal hypoxia; 4) significant vaginal bleeding; and 5) preterm labor with intact membranes.

PPROM was diagnosed based on visual confirmation of amniotic fluid pooling in the posterior vaginal fornix by sterile speculum examination. If uncertainty about amniotic fluid leakage remained after the clinical examination, the presence of insulin-like growth factor-binding protein in the vaginal fluid was assessed (Actim PROM test; Medix Biochemica, Kauniainen, and Finland).

Body fluid samples (amniotic fluid first, cervical fluid second) were collected at the time of admission before the administration of corticosteroids, antibiotics, or tocolytics. Transabdominal amniocentesis to obtain an amniotic fluid sample is a standard part of the department’s clinical management of women with PPROM to assess the intra-amniotic environment. Women with PPROM received corticosteroids to accelerate lung maturation and intravenous antibiotics. Women with intra-amniotic inflammation received clarithromycin for 7 days unless delivery occurred. Those without intra-amniotic inflammation were treated with benzylpenicillin for 7 days unless delivery occurred. In the case of penicillin allergy, women were treated with clindamycin for 7 days unless delivery occurred. Once the final results regarding microbial invasion of the amniotic cavity from cultivation or PCR were known, the attending clinician modified the women’s antibiotic therapies accordingly. Tocolysis was used only in women who developed regular uterine activity during the course of corticosteroids or within 24 h after their administration. The women were managed expectantly, except those with intra-amniotic infection (the presence of both microbial invasion of the amniotic cavity and intra-amniotic inflammation) beyond the 28^th^ gestational week. This subset of women with PPROM was managed actively (labor was induced or an elective cesarean section was performed after finalizing corticosteroid treatment within 72 h of membrane rupture).

This study was approved by the institutional review board of the University Hospital Hradec Kralove (June 2014, No. 201408 S07P). All study participants were Caucasian, and informed consent was obtained from all participants.

Amniotic fluid samples, cervical fluid samples, and the clinical and demographic data of some women from this cohort were used in our previous studies (Musilova et al., 2017a; Musilova et al., 2017b; Hornychova et al., 2018; Kacerovsky et al., 2018; Musilova et al., 2018; Janku et al., 2019; Kacerovsky et al., 2020a; Kacerovsky et al., 2020b; Musilova et al., 2020; Soucek et al., 2020; Spacek et al., 2020; Kacerovsky et al., 2021a; Kacerovsky et al., 2021b; Matulova et al., 2021; Musilova et al., 2021; Stranik et al., 2021)

### Amniotic Fluid Sampling

Ultrasonography-guided transabdominal amniocentesis was performed before administration of corticosteroids, antibiotics, or tocolytics. The details of amniotic fluid sampling have been previously described (Kacerovsky et al., 2020a; Stranik et al., 2021).

### Cervical Fluid Sampling

Cervical fluid samples were collected using Dacron polyester swabs. The details of the procedure have been described previously. Pellets were used to assess the bacterial DNA and *Ureaplasma* spp. DNA (Musilova et al., 2016; Kacerovsky et al., 2020a; Kacerovsky et al., 2021c).

### Assessment of IL-6

Amniotic fluid samples obtained from May 2015 to November 2018 were assessed using a Milenia QuickLine IL-6 lateral flow immunoassay and Milenia POC-Scan Reader (Milenia Biotec, GmbH, Giessen, Germany) (Kacerovsky et al., 2014). The measurement range was 50–10,000 pg/ml. Samples obtained between December 2018 and May 2021 were evaluated using an automated electrochemiluminescence immunoassay method with a Cobas e602 immunoanalyzer, which is part of the Cobas 8,000 platform (Roche Diagnostics, Basel, Switzerland) (Musilova et al., 2020). The measurement range was 1.5–5,000 pg/ml, which could be extended to 50,000 pg/ml with a 10-fold dilution of the sample.

### Detection of *Ureaplasma* spp., *Mycoplasma hominis*, and *Chlamydia trachomatis* in the amniotic fluid.

To assess the amniotic fluid, a commercial AmpliSens® *C. trachomatis*/*Ureaplasma*/*M. hominis*-FRT kit (Federal State Institution of Science, Central Research Institute of Epidemiology, Moscow, Russia) was used to detect DNA from *Ureaplasma* spp., *M. hominis*, and *Ch. trachomatis* in a single PCR tube for each fluid. The details of this procedure have been described previously (Kacerovsky et al., 2020a; Kacerovsky et al., 2020b; Stranik et al., 2021).

### Detection of bacteria other than *Ureaplasma* spp., *Mycoplasma hominis*, or *Chlamydia trachomatis* in the amniotic fluid.

The detection of bacteria other than *Ureaplasma* spp., *M. hominis*, and *Ch. trachomatis* in the women’s amniotic fluid using aerobic/anaerobic cultivation and non-cultivation methods has been described previously (Kacerovsky et al., 2020a; Kacerovsky et al., 2020b; Stranik et al., 2021).

### Detection of *Ureaplasma* spp. and *Mycoplasma hominis* in the Cervical Fluid

To assess the cervical fluid, a commercial AmpliSens® *C. trachomatis*/*Ureaplasma*/*M. hominis*-FRT kit (Federal State Institution of Science, Central Research Institute of Epidemiology, Moscow, Russia) was used to detect DNA from *Ureaplasma* spp., *M. hominis*, and *Ch. trachomatis* in a single PCR tube for each fluid. The details of this procedure have been described previously. The load of *Ureaplasma* spp. (copies/ml) was determined using an absolute quantification technique with an external calibration curve. Plasmid DNA (pCR3, Invitrogen, Carlsbad, CA, United States) was used to prepare a calibration curve.

The relative abundance of *Ureaplasma* spp. DNA in the cervical fluid and total bacterial DNA detection were performed using quantitative RT-PCR–BactQuant (Liu et al., 2012). To quantify the bacterial load, we used the forward primer CCTACGGGDGGCWGCA, reverse primer GGA CTACHVGGGTMTCTAATC, and hydrolysis probe FAM-BHQ1 CAGCCGCGGTA. A calibration curve was generated using 10-fold dilutions of linearized and normalized plasmids containing the cloned target sequence of the 466-bp region in the V3-V4 domain of 16S rRNA at a concentration of 10^7^ copies/µl (Generi Biotech, Hradec Kralove, Czech Republic) (Kacerovsky et al., 2019). The relative abundance of *Ureaplasma* spp. in the cervical microbiota was calculated [(*Ureaplasma* spp. DNA load/total bacterial DNA load) × 100] and expressed as percentages. The PCR conditions used in the BactQuant assay were the same as those used for *Ureaplasma* spp.

### Clinical Definitions

Microbial invasion of the amniotic cavity was determined based on a positive PCR analysis for *Ureaplasma* spp.*, M. hominis, or Ch. trachomatis* in the amniotic fluid, their combination, positivity for the 16S rRNA gene in the amniotic fluid, aerobic/anaerobic cultivation of the amniotic fluid, or a combination of these parameters. Intra-amniotic inflammation was defined as a concentration of IL-6 in the amniotic fluid that was ≥745 pg/ml when measured using a lateral flow immunoassay point-of-care test (Chaemsaithong et al., 2016a; Chaemsaithong et al., 2016b) or ≥3,000 pg/ml when measured using an automated electrochemiluminescence immunoassay method (Musilova et al., 2020). Intra-amniotic infection was defined as the presence of microbial invasion of the amniotic cavity and intra-amniotic inflammation. Women with intra-amniotic infection were further subdivided into those with and without *Ureaplasma* spp. in the amniotic fluid. Sterile intra-amniotic inflammation was defined as the presence of intra-amniotic inflammation without microbial invasion into the amniotic cavity. Colonization of the amniotic cavity was defined as the presence of microbial invasion in the amniotic cavity in the absence of intra-amniotic inflammation. Women with colonization were further subdivided into those with and without *Ureaplasma* spp. in the amniotic fluid. Negative amniotic fluid was defined as amniotic fluid without microbial invasion of the amniotic cavity or intra-amniotic inflammation.

### Statistical Analyses

The demographic and clinical characteristics of the patients were compared using the non-parametric Kruskal–Wallis test for continuous variables and chi-square test for categorical variables, and the results are presented as medians (interquartile range [IQR]) and numbers (%), respectively. The normality of the data was tested using the Anderson–Darling test. The non-parametric Kruskal–Wallis or Mann-Whitney *U* tests were used, as appropriate, to compare the loads of bacteria and *Ureaplasma* spp. DNA in the cervical fluid and the relative abundance of *Ureaplasma* spp. DNA in the cervical fluid. Chi-squared or Fisher’s exact tests were used, as appropriate, to compare the prevalence of *Ureaplasma* spp. DNA in the cervical fluid. Differences were considered statistically significant at *p* < 0.05. All *p*-values were determined using two-tailed tests, and all statistical analyses were performed using GraphPad Prism 8.4.3 for Mac OS X (GraphPad Software, San Diego, CA, United States).

## Results

Overall, 217 women with singleton pregnancies and PPROM between gestational ages 24 + 0 and 33 + 6 weeks were included in the study. Intra-amniotic infection, sterile intra-amniotic inflammation, colonization of the amniotic cavity, and negative amniotic fluid were observed in 20% (44/217), 11% (24/217), 10% (21/217), and 59% (128/217) of women, respectively. The demographic and clinical data of the women with PPROM, as well as short-term neonatal outcomes are summarized in Table 1.

**TABLE 1:** Demographical and clinical characteristics and short-term neonatal outcomes of pregnancies with preterm prelabor rupture of membranes prior to 34 weeks of gestation according to the presence of intra-amniotic infection, sterile intra-amniotic inflammation, colonization of the amniotic cavity, and negative amniotic fluid.

Characteristic	Intra-amniotic infection (*n* = 44)	Sterile intra-amniotic inflammation (*n* = 24)	Colonization of the amniotic cavity (*n* = 21)	Negative amniotic fluid (*n* = 128)	*p-*value
Maternal age [years, median (IQR)]	31 (25–36)	31 (29–36)	34 (28–37)	31 (27–34)	0.42
Primiparous [number (%)]	21 (48%)	7 (29%)	7 (33%)	74 (58%)	**0.02**
Smoking [number (%)]	7 (16%)	3 (13%)	2 (10%)	18 (14%)	0.91
Pre-pregnancy body mass index [kg/m^2^, median (IQR)]	23.3 (20.4–27.3)	25.5 (20.6–28.9)	23.6 (20.4–26.2)	24.3 (21.4–28.6)	0.40
Gestational age at sampling [weeks + days, median (IQR)]	28 + 5 (25+2–31 + 5)	26 + 6 (24+0–30 + 1)	31 + 5 (31+0–33 + 0)	31 + 5 (30+0–33 + 1)	**<0.0001**
Gestational age at delivery [weeks + days, median (IQR)]	29 + 4 (26+4–32 + 0)	30 + 0 (27+6–32 + 2)	32 + 5 (31+4–33 + 6)	32 + 5 (30+6–33 + 5)	**<0.0001**
Latency from PPROM to amniocentesis [hours, median (IQR)]	4 (3–10)	5 (4–13)	5 (2–12)	5 (3–9)	0.87
Latency from amniocentesis to delivery [days, median (IQR)]	76 (42–169)	188 (52–632)	96 (65–159)	79 (22–285)	0.15
Presence of *Ureaplasma* spp. in amniotic fluid [number (%)]	26 (59%)	0 (0%)	15 (71%)	0 (0%)	**<0.0001**
CRP levels at admission [mg/L, median (IQR)]	18.0 (4.9–36.5)	6.4 (2.5–9.2)	3.2 (1.5–7.0)	4.8 (2.9–8.9)	**<0.0001**
WBC count at admission [× 10^9^ L, median (IQR)]	15.4 (11.2–18.6)	12.1 (10.0–15.2)	12.2 (10.2–14.4)	12.6 (10.5–15.4)	**0.02**
Administration of corticosteroids [number (%)]	42 (96%)	22 (92%)	20 (95%)	125 (98%)	0.52
Administration of antibiotics [number (%)]	44 (100%)	24 (100%)	21 (100%)	127 (99%)	0.87
Spontaneous vaginal delivery [number (%)]	23 (52%)	11 (46%)	13 (62%)	75 (59%)	0.42
Cesarean section [number (%)]	11 (25%)	13 (54%)	7 (33%)	53 (41%)	0.35
Forceps delivery [number (%)]	0 (0%)	0 (0%)	1 (5%)	0 (0%)	**0.03**
Birth weight [grams, median (IQR)]	1285 (820–1873)	1345 (983–1733)	1910 (1715–2050)	1915 (1563–2198)	**<0.0001**
Apgar score <7; 5 min [number (%)]	7 (16%)	2 (8%)	0 (0%)	4 (3%)	**0.01**
Apgar score <7; 10 min [number (%)]	3 (7%)	0 (0%)	0 (0%)	2 (2%)	0.15
Transient tachypnea of newborns [number (%)]	2 (5%)	1 (4%)	1 (5%)	14 (11%)	0.12
Respiratory distress syndrome [number (%)]	29 (65%)	14 (58%)	4 (19%)	43 (34%)	**<0.0001**
Bronchopulmonary dysplasia [number (%)]	15 (34%)	5 (21%)	0 (0%)	7 (6%)	**<0.0001**
Need for intubation [number (%)]	5 (11%)	2 (8%)	1 (5%)	4 (3%)	**0.03**
Intraventricular hemorrhage (grades I-II) [number (%)]	5 (11%)	5 (21%)	5 (24%)	24 (19%)	0.38
Intraventricular hemorrhage (grades III-IV) [number (%)]	3 (7%)	0 (0%)	0 (0%)	0 (0%)	**0.003**
Retinopathy of prematurity [number (%)]	5 (11%)	4 (17%)	0 (0%)	2 (2%)	**0.001**
Necrotizing enterocolitis [number (%)]	2 (5%)	1 (4%)	0 (0%)	1 (1%)	0.08
Early-onset sepsis [number (%)]	5 (11%)	2 (8%)	2 (10%)	1 (1%)	**0.002**
Late-onset sepsis [number (%)]	5 (11%)	0 (0%)	0 (0%)	0 (0%)	**0.0001**
Compound neonatal morbidity [number (%)]	31 (70%)	17 (71%)	8 (38%)	70 (56%)	**0.04**
Neonatal death [number (%)]	2 (5%)	1 (4%)	0 (0%)	2 (2%)	0.21

Abbreviations; CRP, C-reactive protein; IQR, interquartile range; PPROM, preterm prelabor rupture of membranes; WBC, white blood cells

Continuous variables were compared using a nonparametric Kruskal–Wallis test. Categorical variables were compared using chi-square test. Statistically significant results are marked in bold. Continuous variables are presented as median (interquartile range) and categorical as number (%).

The most common microorganisms in the amniotic fluid were *Ureaplasma* spp., which were found in 59% (26/44) and 71% (15/21) of women with intra-amniotic infection and colonization of the amniotic cavity, respectively. All microbial findings in the amniotic fluid that were identified in the women with intra-amniotic infection are shown in Table 2.

**TABLE 2:** The microbial species identified in the amniotic fluid from pregnancies with preterm prelabor rupture of membranes prior to 34 weeks of gestation complicated with intra-amniotic infection and colonization of the amniotic cavity.

Intra-amniotic infection (*n* = 44)	Colonization of the amniotic cavity (*n* = 21)
*Atopobium vaginae, Dialister micraerophilus, Fusobacterium nucleatum, Ureaplasma* spp. (*n* = 1)	*Corynebacterium tuberculosteraricum, Dermabacter hominis, Staphylococcus epidermidis* (*n* = 1)
*Aerococcus christensenii, Gardnerella vaginalis, Ureaplasma* spp. (*n* = 1)	*Gardnerella vaginalis, Ureaplasma* spp. (*n* = 1)
*Campylobacter ureolyticus, Streptococcus anginosus, Streptococcus oralis* (*n* = 1)	*Mycoplasma hominis, Ureaplasma* spp. (*n* = 1)
*Chlamydia trachomatis, Ureaplasma spp.* (*n* = 2)	*Ureaplasma* spp. (*n* = 13)
*Fusobacterium nucleatum, Ureaplasma spp.* (*n* = 1)	*Escherichia coli* (*n* = 1)
*Streptococcus anginosus, Ureaplasma* spp. (*n* = 1)	*Gardnerella vaginalis* (*n* = 1)
*Ureaplasma* spp. (*n* = 20)	*Lactobacillus iners* (*n* = 1)
*Haemophilus influenzae* (*n* = 5)	*Sneathia sanguinegens* (*n* = 1)
*Anaerococcus tetradius* (*n* = 1)	*Streptococcus agalactiae* (*n* = 1)
*Enterococcus faecalis* (*n* = 1)	
*Fusobacterium nucleatum* (*n* = 1)	
*Gardnerella vaginalis* (*n* = 1)	
*Lactobacillus jensenii* (*n* = 1)	
*Parvimonas micra* (*n* = 1)	
*Peptoniphilus spp.* (*n* = 1)	
*Sneathia sanguinegens* (*n* = 1)	
*Streptococcus agalactiae* (*n* = 1)	
*Streptococcus anginosus* (*n* = 1)	
*Streptococcus intermedius* (*n* = 1)	
Non-identifiable bacteria by sequencing (*n* = 1)	

*Ureaplasma* spp. DNA in the cervical fluid was identified in 61% (133/217) of the women. All of the women with *Ureaplasma* spp. DNA in their amniotic fluid (*n* = 41) also had *Ureaplasma* spp. DNA in their cervical fluid.

### Prevalence of *Ureaplasma* spp. DNA in the Cervical Fluid

**FIGURE 1:**
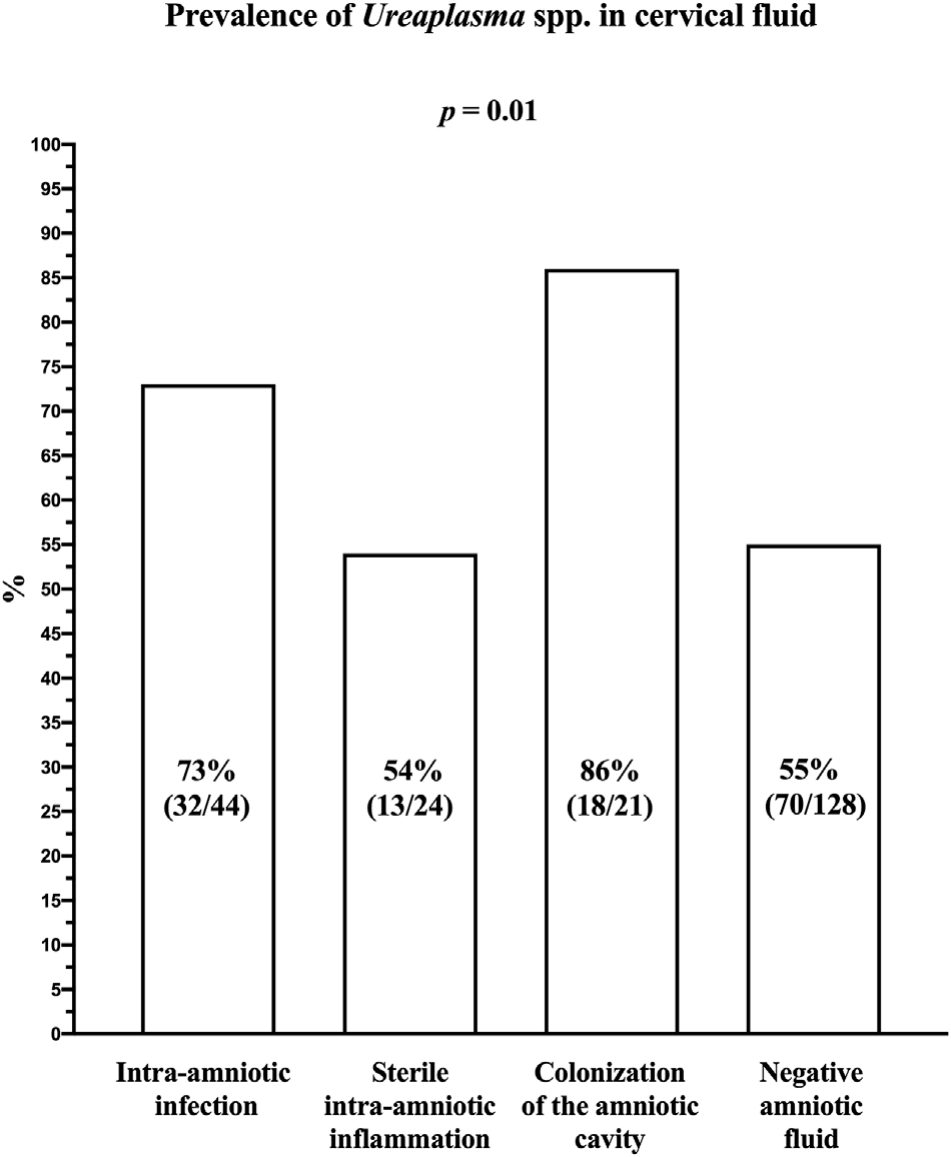
Prevalence of *Ureaplasma* spp. DNA in the cervical fluid of women with preterm prelabor rupture of membranes before 34 weeks of gestation according to the presence of intra-amniotic infection, sterile intra-amniotic inflammation, colonization of the amniotic cavity, and negative amniotic fluid.

The prevalence of *Ureaplasma* spp. DNA in the cervical fluid varied among the subgroups of women with intra-amniotic infection (73% [32/44]), sterile intra-amniotic inflammation (54% [13/24]), colonization (86% [18/21]), and negative amniotic fluid (55% [70/128]; *p* = 0.01; Figure 1).

Women with negative amniotic fluid had a lower prevalence of *Ureaplasma* spp. DNA in their cervical fluid compared to the women with intra-amniotic infections (*p* = 0.05) and colonization (*p* = 0.008); however, they had a similar prevalence to those with sterile intra-amniotic inflammation (*p* = 1.00).

### Microbial Load of *Ureaplasma* spp. DNA in the Cervical Fluid

**FIGURE 2:**
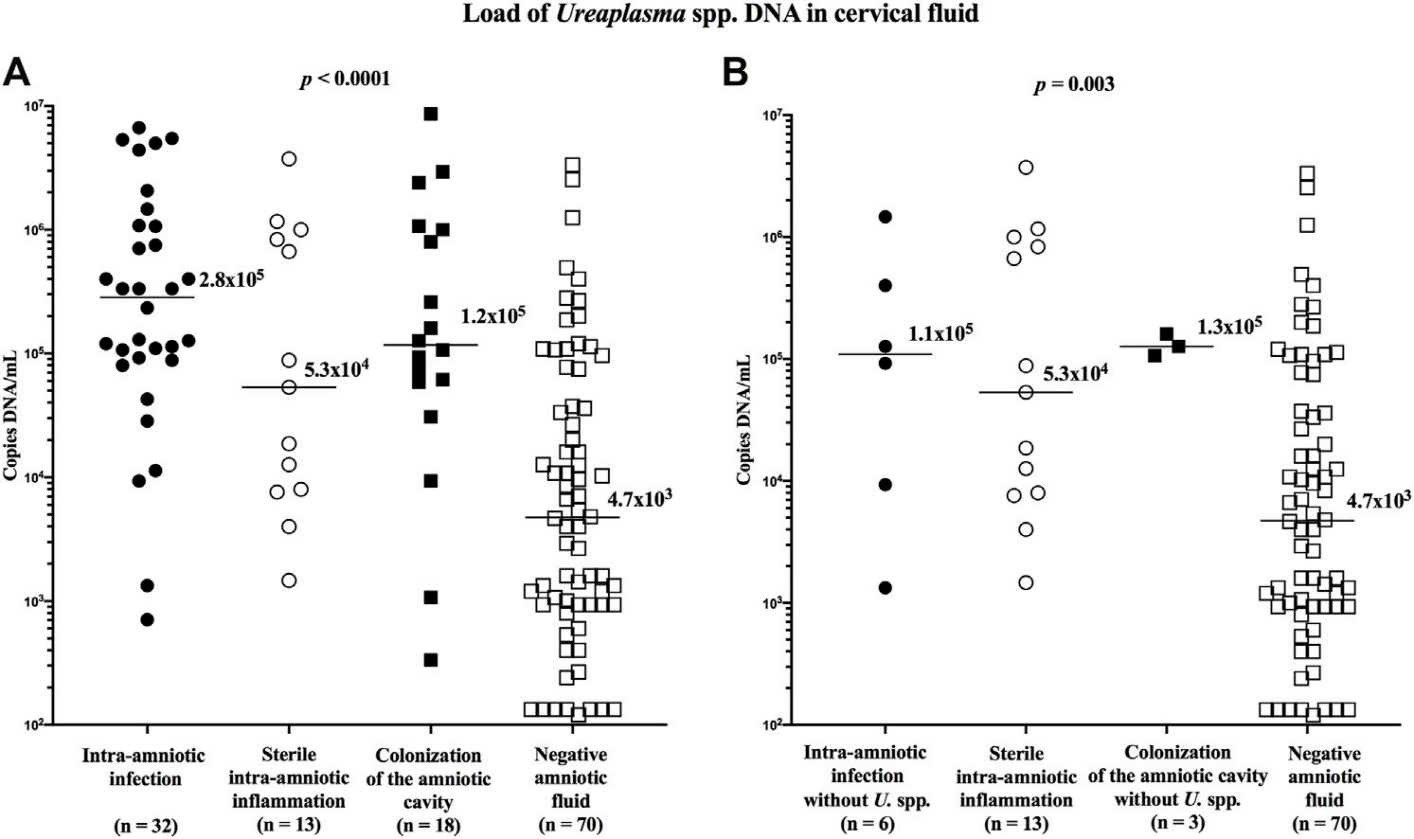
Comparison of the loads of *Ureaplasma* spp. DNA in the cervical fluid among groups of the women with intra-amniotic infection, sterile intra-amniotic inflammation, colonization of the amniotic fluid, and negative amniotic fluid **(A)** and among the same groups when women with the presence of *Ureaplasma* spp. in amniotic fluid were excluded **(B)**. The median values are marked.

The load of *Ureaplasma* spp. DNA in the cervical fluid varied among the subgroups (median [IQR]; intra-amniotic infection: 2.8 × 10^5^ copies DNA/mL [8.9 × 10^4^–1.1 × 10^6^]; sterile intraamniotic inflammation: 5.3 × 10^4^ copies DNA/mL [7.8 × 10^3^–9.2 × 10^5^]; colonization 1.2 × 10^5^ copies DNA/ml [5.2 × 10^4^–1.1 × 10^6^]; and negative amniotic fluid: 4.7 × 10^3^ copies DNA/mL [9.3 × 10^2^–4.6 × 10^4^]; *p* < 0.0001; Figure 2A), as well as when the women with *Ureaplasma* spp. in the amniotic fluid were excluded (median [IQR]; intra-amniotic infection: 1.1 × 10^5^ copies DNA/mL [7.3 × 10^3^–6.7 × 10^5^]; colonization 1.3 × 10^5^ copies DNA/mL [1.1 × 10^5^–1.6 × 10^5^]; *p* = 0.003; Figure 2B).

Women with negative amniotic fluid had a lower load of *Ureaplasma* spp. DNA in the cervical fluid compared to those with intra-amniotic infection (*p <* 0.0001; without *Ureaplasma* spp. in amniotic fluid: *p =* 0.04), sterile intra-amniotic inflammation (*p =* 0.004), and colonization (*p* = 0.0001; without *Ureaplasma* spp. in amniotic fluid: *p =* 0.04).

### Relative Abundance of *Ureaplasma* spp. in the Cervical Fluid

**FIGURE 3:**
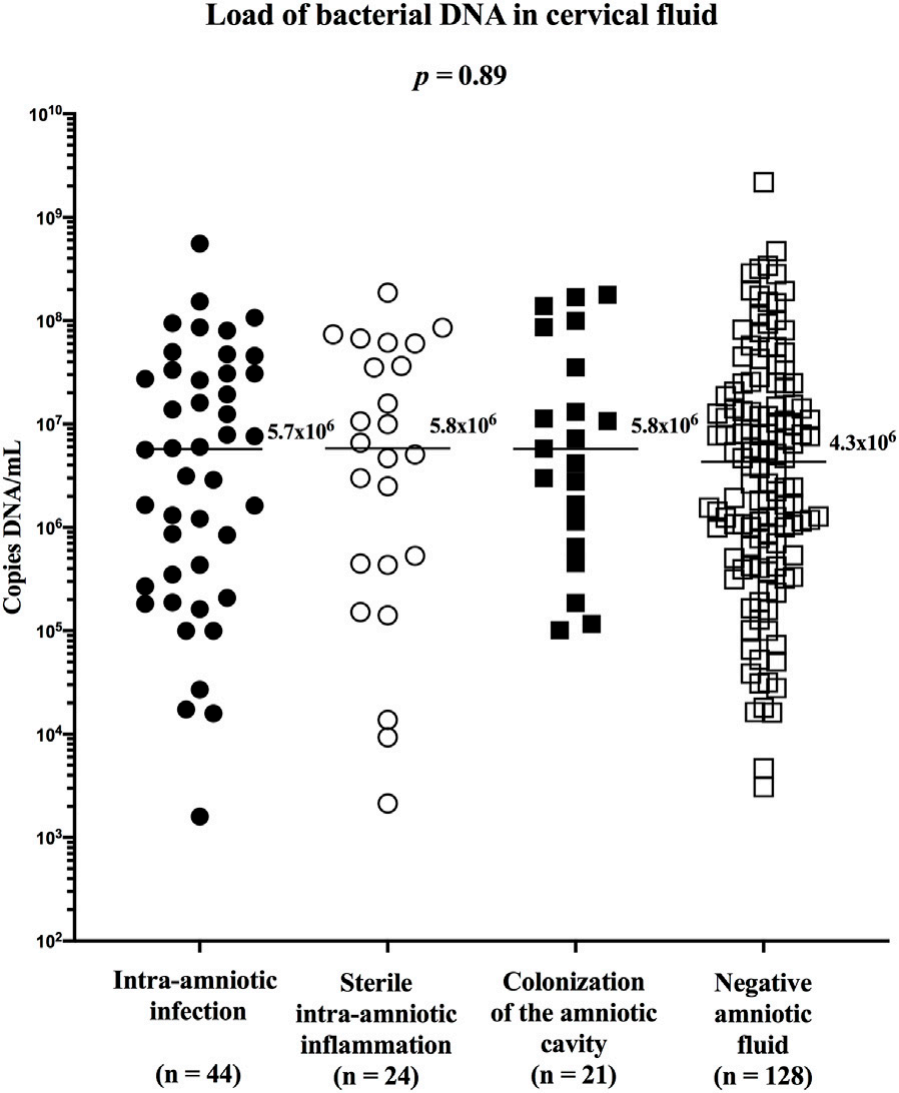
Comparison of the load of bacterial DNA in the cervical fluid among groups of women with intra-amniotic infection, sterile intra-amniotic inflammation, colonization of the amniotic fluid, and negative amniotic fluid.

To assess the relative abundance of *Ureaplasma* spp. in the cervical fluid between the subgroups of women with PPROM, the amount of bacterial DNA in the cervical fluid was first evaluated. No difference in the concentration of bacterial DNA was identified between the subgroups (median [IQR]; intra-amniotic infection: 5.7 × 10^6^ copies DNA/ml [2.9 × 10^5^–3.1 × 10^7^]; sterile intra-amniotic inflammation: 5.8 × 10^6^ copies DNA/ml [4.4 × 10^5^–5.4 × 10^7^]; colonization: 5.8 × 10^6^ copies DNA/ml [9.0 × 10^5^–6.1 × 10^7^]; and negative amniotic fluid: 4.3 × 10^6^ copies DNA/ml [5.9 × 10^5^–2.0 × 10^7^]; *p* = 0.89; Figure 3).

**FIGURE 4:**
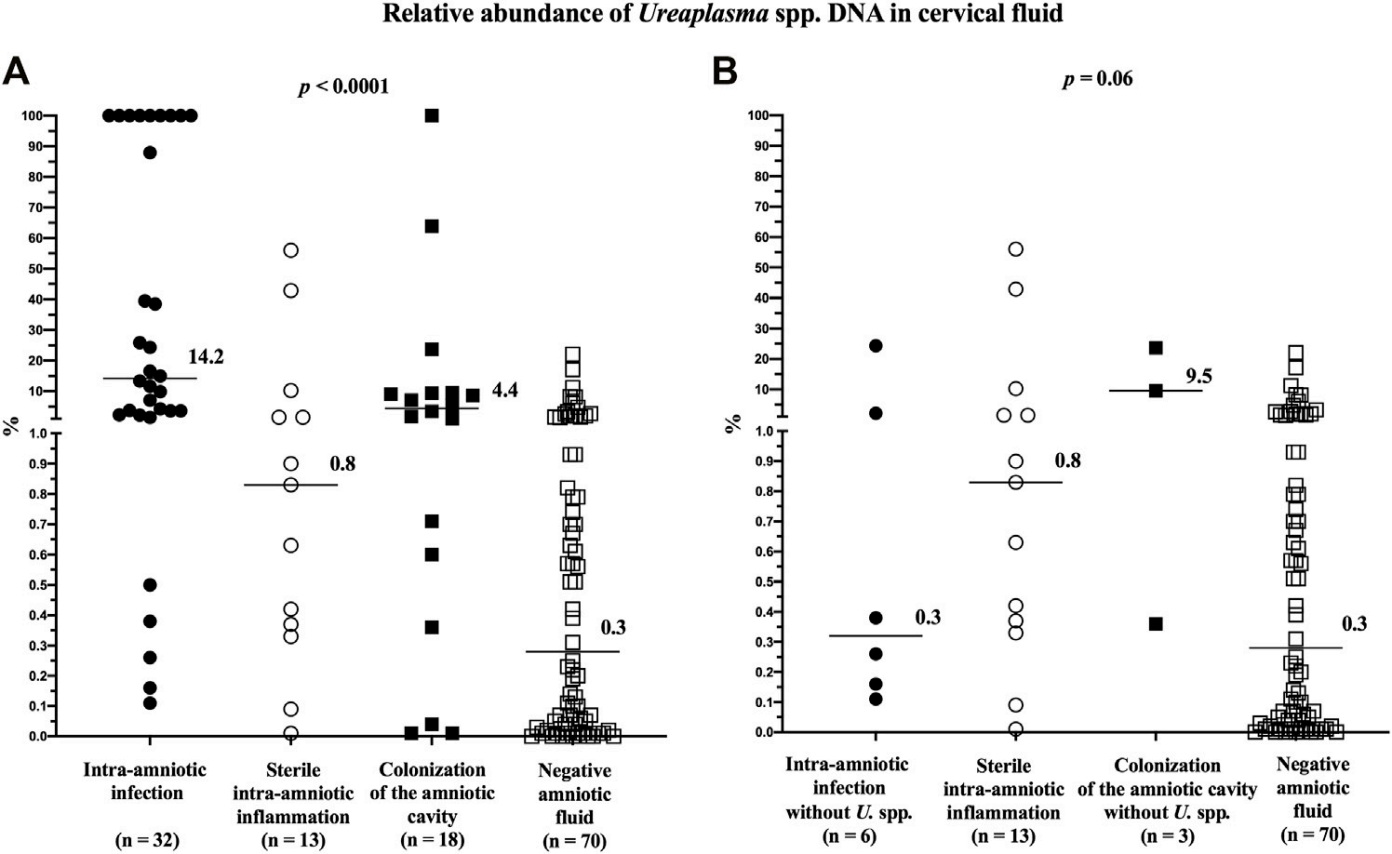
Comparison of the relative abundance of *Ureaplasma* spp. DNA in the cervical fluid among groups of women with intra-amniotic infection, sterile intra-amniotic inflammation, colonization of the amniotic fluid, and negative amniotic fluid **(A)** and among the same groups when women with the presence of *Ureaplasma* spp. in their amniotic fluid were excluded **(B)**. The median values are marked.

The relative abundance of *Ureaplasma* spp. in the cervical fluid differed among the subgroups (median [IQR]; intra-amniotic infection: 14.2% [2.6–100.0]; sterile intra-amniotic inflammation: 0.8% [0.4–5.9]; colonization: 4.4% [0.5–9.4]; and negative amniotic fluid: 0.3% [0.1–0.9]; *p* < 0.0001; Figure 4A). However, after excluding the women with *Ureaplasma* spp. in the amniotic fluid, the difference reached borderline statistical significance (median [IQR]; intra-amniotic infection: 0.3% [0.2–7.7]; colonization: 9.5% [0.4–23.7]; *p* = 0.06; Figure 4B).

Women with negative amniotic fluid had a lower relative abundance than those with intra-amniotic infection (*p* < 0.0001), sterile intra-amniotic inflammation (*p* = 0.05), or colonization (*p* = 0.008). After excluding the women with *Ureaplasma* spp. in the amniotic fluid, there was no difference (intra-amniotic infection: *p* = 0.36; colonization: *p* = 0.06).

## Discussion

The principal findings of this study in women with PPROM before 34 weeks were as follows: 1) the total prevalence of *Ureaplasma* spp. DNA in the cervical fluid was 61%; 2) the presence of intra-amniotic infection and colonization was related to higher rates of *Ureaplasma* spp. DNA in the cervical fluid compared to those with negative amniotic fluid results; and 3) the presence of intra-amniotic infection, sterile intra-amniotic inflammation, or colonization of the amniotic fluid was associated with higher microbial loads of *Ureaplasma* spp. DNA in the cervical fluid compared to those with negative amniotic fluid; and 4) the presence of sterile intra-amniotic inflammation was related to a higher relative abundance of *Ureaplasma* spp. DNA in the cervical fluid compared to that in negative amniotic fluid.

*Ureaplasma* spp. are considered commensal microorganisms of the cervical/vaginal niche owing to: 1) their high prevalence in pregnant or non-pregnant women, and 2) the absence of a difference in the prevalence between women of reproductive ages with and without symptoms of infection of the urogenital tract (Marovt et al., 2015; Sweeney et al., 2017). In pregnant women, the presence of *Ureaplasma* spp. in the cervical/vaginal niche has been shown to be associated with adverse pregnancy outcomes (Abele-Horn et al., 2000; Kafetzis et al., 2004; Kataoka et al., 2006). Accordingly, *Ureaplasma* spp. in the cervical/vaginal niche is more frequently observed in pregnancies complicated by PPROM than in uncomplicated pregnancies (Larsen and Hwang, 2010; Donders et al., 2017; Sprong et al., 2020). Its prevalence in PPROM pregnancies varies between 53 and 73% (Kwak et al., 2014; Kwak et al., 2015; Musilova et al., 2016). The observations from this study (61%) are in agreement with previously published findings.

Interestingly, in this study, women with intra-amniotic infection and colonization of the amniotic cavity had higher rates of *Ureaplasma* spp. DNA in their cervical fluid than did women with sterile intra-amniotic inflammation and negative amniotic fluid. In other words, a higher proportion of *Ureaplasma* spp. DNA in the cervical fluid was found in the subgroups of women with microbial invasion of the amniotic cavity. This observation is consistent with the findings of our previous study, in which women with microbial invasion of amniotic inflammation had a higher rate of *Ureaplasma* spp. DNA in the cervical fluid than did those without this complication (Musilova et al., 2016). Notably, in this study, no difference in the frequency of *Ureaplasma* spp. in the cervical fluid between women with sterile inflammation and negative amniotic fluid was identified. This observation differs from that recently reported in women with preterm labor and intact membranes. In that study, the prevalence of *Ureaplasma* spp. DNA in the group of women with sterile intra-amniotic inflammation was comparable to that in women with intra-amniotic infection; however, it was 2-fold higher than that in women with negative amniotic fluid (Kacerovsky et al., 2021c). The diversity in observations between the studies on different phenotypes of spontaneous preterm delivery supports the hypothesis that pathophysiological pathways leading to the development of sterile intra-amniotic inflammation might differ between PPROM and preterm labor with intact membranes.

Abnormal microbiota in the cervical/vaginal niche have been shown to be associated not only with a higher frequency of *Ureaplasma* spp. but also with higher loads of these bacteria in that niche (Donders et al., 2017). Kwak *et al.* reported that a higher load of *Ureaplasma* spp. in the vaginal niche is associated with a higher frequency of histological chorioamnionitis in women with PPROM (Kwak et al., 2014). In line with their report, a difference in the microbial load of *Ureaplasma* spp. DNA in the cervical fluid among the subgroups with intra-amniotic infection, sterile intra-amniotic inflammation, colonization, and negative amniotic fluid (with the lowest levels found in those with negative amniotic fluid) was found in this study. However, this observation was not in concordance with our previous study, where no difference in the load of *Ureaplasma* spp. DNA in the cervical fluid was identified in women with and without microbial invasion of the amniotic cavity (Musilova et al., 2016). The reason for this difference is not fully clear; however, it can be explained by the differences between the studies, including: 1) the sample sizes of women with *Ureaplasma* spp. DNA (previous: *n* = 40; current: *n* = 133) that were studied; and 2) the gestational ages considered during sampling (previous: 24 + 0 to 36 + 6; current: 24 + 0 to 33 + 6).

The relative abundance of *Ureaplasma* spp. DNA in the cervical fluid might represent a more precise tool with which to characterize the association between the composition of the cervical microbiome and intra-amniotic complications, as opposed to the assessment of the absolute number of copies of *Ureaplasma* spp. DNA that are present. In this study, women with PPROM and sterile intra-amniotic inflammation had an approximately 2.5-fold higher relative abundance of *Ureaplasma* spp. DNA compared to women with negative amniotic fluid results. This observation should be considered in terms of the pathophysiology of the development of sterile intra-amniotic inflammation in PPROM.

Taken together, the results from this study suggest that 1) the subgroup of women with PPROM and negative amniotic fluid differs from those with intra-amniotic complications in terms of frequency, loads, and relative abundance of *Ureaplasma* spp. DNA in the cervical fluid; and 2) differences exist between PPROM and preterm labor with intact membranes with regard to the association between *Ureaplasma* spp. DNA in the cervical fluid and intra-amniotic complications. Collectively, these observations further support that pathophysiological pathways leading to the development of intra-amniotic complications might differ between PPROM and preterm labor with intact membranes.

This study has several strengths. First, a relatively large cohort of women with a well-defined clinical phenotype for spontaneous preterm delivery (PPROM) was assessed. Secondly, the presence of *Ureaplasma* spp. DNA in the cervical fluid was evaluated with a specific PCR procedure, which allowed us to identify even very low loads of *Ureaplasma* spp. DNA. Third, thorough assessment of microbial invasion of the amniotic cavity, with the use of a combination of non-specific PCR (16S rRNA) followed by sequencing, specific PCR for *Ureaplasma* spp., *Mycoplasma hominis*, and *Chlamydia trachomatis*, and aerobic/anaerobic cultivation allowed us to precisely dissect the subgroups of women with sterile intra-amniotic inflammation.

This study has limitations that are worth mentioning. First, in women with PPROM, amniotic fluid leaks from the amniotic cavity through the cervix into the posterior vaginal fornix. This means that the leaking amniotic fluid can contaminate the cervical fluid. Therefore, the fluid obtained with a Dacron swab from the endocervical canal of women with PPROM should be considered not just as cervical fluid but as a compound fluid, composed of both cervical fluid and amniotic fluid components. Its composition, as well as the ratio between amniotic and cervical fluids, might vary among women with PPROM and depend on various clinical parameters and scenarios (e.g., amount of residual amniotic fluid, location of the membrane rupture site, position of the fetus, and interval between PPROM and sampling) that occur at the time of sampling. Therefore, if the leaked amniotic fluid contains *Ureaplasma* spp. DNA (in cases of intra-amniotic infection and colonization of the amniotic cavity caused by *Ureaplasma* spp.), it can affect the total microbial load of the *Ureaplasma* spp. DNA and its relative abundance in the fluid obtained from the endocervical canal. Unfortunately, the presence of this phenomenon is inevitable in PPROM and could affect the assessment of microbial loads and the relative abundance of *Ureaplasma* spp. DNA in the cervical fluid among subgroups of women with PPROM. On the other hand, it is highly unlikely that the presence of *Ureaplasma* spp. DNA in leaked amniotic fluid could affect the assessment of the prevalence of *Ureaplasma* spp. in the cervical fluid, since the cervical/vaginal niche is considered to be a primary source of *Ureaplasma* spp.

Second, biovars, serovars, and other types of *Ureaplasma* spp. were not assessed and considered in this study. This fact should be taken as a shortcoming of this study. However, there is evidence of the possibility of horizontal gene transfer in *Ureaplasma* spp., allowing them to carry markers of multiple serovars (Xiao et al., 2011; Sweeney et al., 2017). Thus, the assessment of serovars could have a limited diagnostic value. Third, during the study period, the methods to assess the concentrations of IL-6 in amniotic fluid were modified. Therefore, the concentrations of IL-6 in this study were determined using two different approaches (lateral flow immunoassay point-of-care test and automated electrochemiluminescence). This fact prevents us from assessing the association between the intensity of the intra-amniotic inflammatory response, measured by IL-6 concentrations in amniotic fluid, and the loads of *Ureaplasma* spp. DNA in the cervical fluid.

In conclusion, in PPROM at < 34 weeks, the presence of intra-amniotic infection, sterile intra-amniotic inflammation, or colonization of the amniotic fluid was associated with a higher prevalence and/or load of *Ureaplasma* spp. DNA in cervical fluid than in the absence of intra-amniotic complications.

## Data Availability

The raw data supporting the conclusion of this article will be made available by the authors, without undue reservation.
